# Supplementary material for: Identification of photoperiod-induced specific miRNAs in the adrenal glands of Sunite sheep (Ovis aries)
Source: Front Vet Sci. 2022 Jul 22;9:888207. doi: 10.3389/fvets.2022.888207 (PMC9354845; doi:10.3389/fvets.2022.888207)
Supplement: Supplementary file 1 [file Data_Sheet_1.ZIP › Supplementary file/miRDeep2 file.zip!/miRDeep2 file/Know_Structure/SP42A2/oar-mir-106b.pdf]

miRBase precursor	: oar-mir-106b
Total read count	: 4798
oar-miR-106b read count	: 1819
remaining reads	: 2979

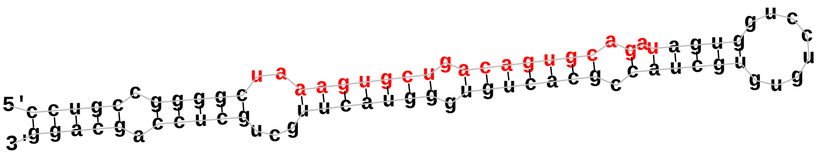

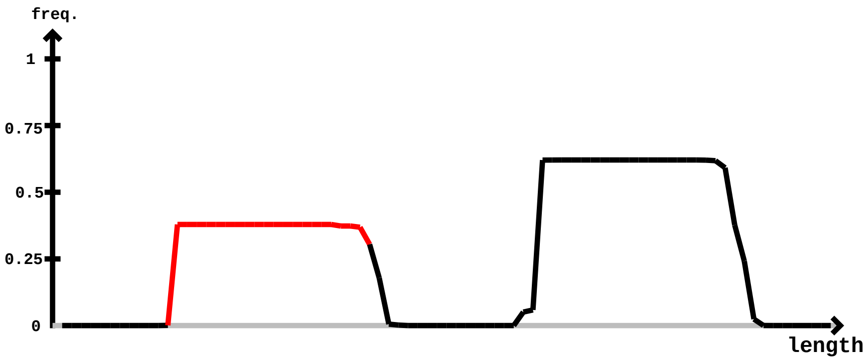


```
                        oar-miR-106b
5'- ccugccggggcuaaagugcugacagugcagauagugguccugugugcuaccgcacuguggguacuugcugcuccagcagg   -3'    exp
    (((((.(((((..(((((((.(((((((.(.(((((........)))))).))))))).)))))))...))))).)))))    reads    mm    sample
    ..........cuaaagugcugacagugcagau................................................    1        0     seq
    ..........cuaaagugcugacagugcagaua...............................................    3        0     seq
    ...........uaaUgugcugacagugc....................................................    1        1     seq
    ...........uaaagugcugacagugA....................................................    3        1     seq
    ...........uaaagugcugacagugc....................................................    23       0     seq
    ...........uaaagugcugacaguUc....................................................    1        1     seq
    ...........uaaagugcugacagugcag..................................................    22       0     seq
    ...........uaaagugcugacagugcaga.................................................    307      0     seq
    ...........uaaagugcugacagugcagau................................................    604      0     seq
    ...........uaaagugcugacagugcagaua...............................................    828      0     seq
    ...........uaaagugcugacagugcagauag..............................................    17       0     seq
    ...........uaaagugcugacagugcagauagu.............................................    9        0     seq
    ...............................................uaccgcacuguggguacuu..............    2        0     seq
    ...............................................uaccgcacuguggguacuug.............    1        0     seq
    ...............................................uaccgcacuguggguacuugc............    21       0     seq
    ...............................................uaccgcacuguggguacuugcu...........    216      0     seq
    ...............................................uaccgcacuguggguacuugcug..........    2        0     seq
    ...............................................uaccgcacuguggguacuugcugc.........    1        0     seq
    ................................................accgcacuguggguacuugc............    5        0     seq
    ................................................accgcacuguggguacuugcu...........    17       0     seq
    ................................................accgcacuguggguacuugcug..........    12       0     seq
    ................................................accgcacuguggguacuugcugc.........    4        0     seq
    .................................................ccgcacuguggguacuu..............    1        0     seq
    .................................................ccgcacuguggguacuug.............    9        0     seq
    .................................................ccgcacuguggguacuugc............    101      0     seq
    .................................................ccgcacuguggguacuugcu...........    795      0     seq
    .................................................ccgcacuguggguacuugcug..........    636      0     seq
    .................................................ccgcacuguggguacuugcugc.........    1038     0     seq
    .................................................ccgcacuguggguacuugcugcu........    115      0     seq
    .................................................ccgcacuguggguacuugcugcuc.......    1        0     seq
    ..................................................cgcacuguggguacuugcu...........    1        0     seq
    ...................................................gcacuguggguacuugcugc.........    1        0     seq
```
